# Whole Genome Sequence of the gut commensal protist *Tritrichomonas musculus* isolated from laboratory mice

**DOI:** 10.1038/s41597-025-04921-0

**Published:** 2025-04-08

**Authors:** Eliza V. C. Alves-Ferreira, Madeline R. Galac, Hernan A. Lorenzi, Margaret C. W. Ho, Erick T. Tjhin, Ana Popovic, John Parkinson, Michael E. Grigg

**Affiliations:** 1 Laboratory of Parasitic Diseases, NIAID, National Institutes of Health, Bethesda, Maryland USA; 2 Bioinformatics and Computational Biology Branch, NIAID, National Institutes of Health, Bethesda, Maryland USA; 3 Program in Molecular Medicine, The Hospital for Sick Children, Toronto, Ontario Canada; 4 Department of Biochemistry, University of Toronto, Toronto, Ontario Canada; 5 Department of Molecular Genetics, University of Toronto, Toronto, Ontario Canada

**Keywords:** Parasite genomics, Whole genome amplification

## Abstract

*Tritrichomonas musculus* is a commensal protist colonizing the large intestine of laboratory mice. Parasite colonization reshapes the gut microbiome and modulates mucosal immunity. This parasite is refractory to axenic culture. In order to facilitate functional genomic investigations we assembled a 193.49 Mbp high quality reference genome from FACS-purified parasites recovered from monocolonized mice using an integrated approach that combined long-read (PacBio and Oxford Nanopore) sequencing technologies for the draft genome assembly. The genome assembled into 756 contigs and RNA-Seq data was used to support the gene models for 46,131 annotated genes. Of these, 24,215 genes had an InterPro, Enzyme Commission and/or a Gene Ontology annotation. BUSCO analyses established that 53% of the genome annotations matched with available BUSCO genes in the eukaryote_odb10 database. This high quality reference genome will serve as a valuable resource to develop a metabolic and genetic model to grow *T. musculus* axenically and study genes relevant to its biology, life cycle transmission, and pathogenesis.

## Background & Summary

Commensal bacteria, fungi, viruses and protists are common within the mammalian intestine, interacting with each other and contributing to gut homeostasis^1^. Protozoa are increasingly being recognised as essential to the gut microenvironment^2,3^ by their ability to reshape the constituent bacteriome^4,5^ and mucosal immune responses^6–8^ with minimal signs of disease. Despite the biological significance of these commensal protists, reference genome assemblies with annotation to facilitate genetic diversity and comparative genomic studies are often lacking.

**Fig. 1:**
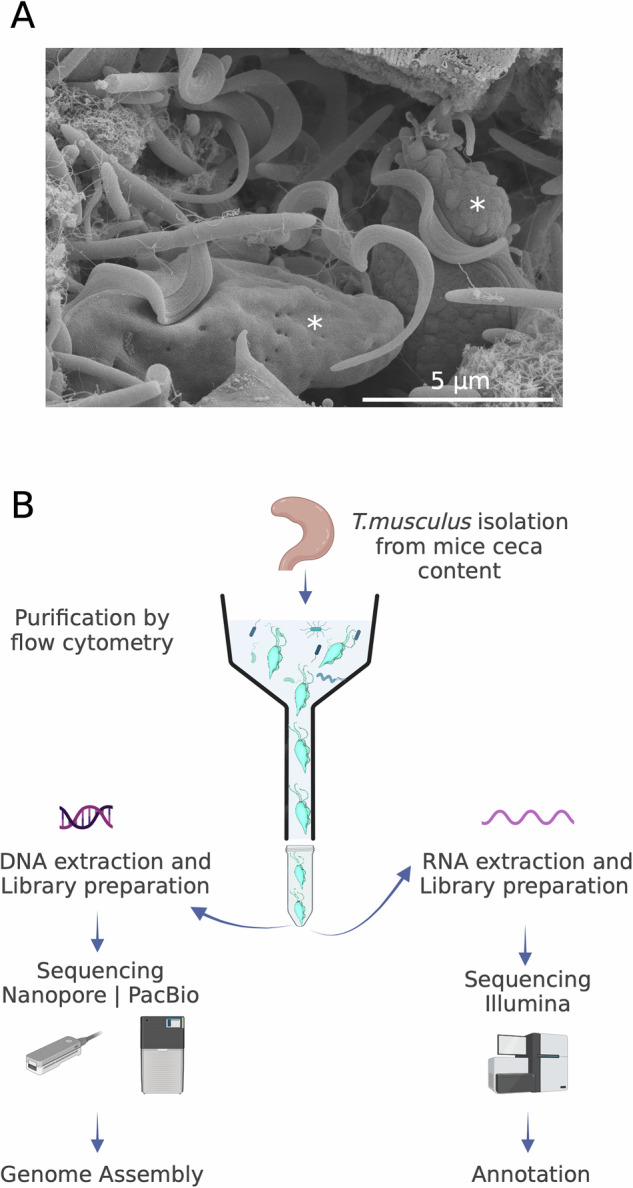
(**A**) Representative *T. musculus* trophozoite colonizing mouse ceca surrounded by bacteria. *In situ* cecum scanning electron microscopy image (methods available in Tuzlak & Alves-Ferreira, 2023). (**B**) Schematic workflow for *T. musculus* purification and whole genome sequencing. Created with BioRender.com.

*Tritrichomonas musculus*, also known as *Tmu*, is an extracellular anaerobic commensal protist that frequently colonizes the mouse large intestine. The trophozoites have an ovoid shape, are 10 to 16 µm long, possess an anterior and posterior flagella and one undulating membrane^2^ (Fig. 1A). Mice colonized with *T. musculus* do not present with disease, including symptoms such as diarrhea and weight loss, but do exhibit a mild goblet cell hyperplasia, host epithelial cell inflammasome activation, including the release of IL-1β and IL-18, and a profound shift in the 16S bacterial community structure during *T. musculus* colonization^5^. This reprogramming of the constituent microbiome and host immune potential is sufficient to protect mice from a lethal challenge with the pathogenic bacteria *Salmonella typhimurium*^6^. However, the change in the immune status increases the risk of colitis and colorectal cancer^6,7^.

Here, we describe a high quality, annotated *T. musculus* genome assembly. We purified *T. musculus* from the cecal content of laboratory mice monocolonized with the EAF2021 isolate. The protists were purified by Percoll gradient centrifugation followed by flow cytometry prior to DNA and RNA extraction. Sequencing libraries were prepared using purified DNA or RNA and Illumina short-read, PacBio and Oxford Nanopore (ONT) long-read sequencing was performed to facilitate genome assembly and annotation (Fig. 1B). The genome is 193.5 Mb-long with an N50 scaffold length of 3.5 Mb that assembled into 756 contigs. A total of 46,131 protein-coding genes were identified. This annotated genome will be a useful resource to develop a genetic model system to study the biology, evolution and diversity of *T. musculus* and to discover the metabolic pathways that are essential for colonization and survival within the host.

## Methods

### Sample collection, whole genome DNA and RNA sequencing

*Tritrichomonas musculus* trophozoites of the EAF2021 isolate were purified from laboratory mice cecal content using flow cytometry as described previously^2^. High molecular weight (HMW) genomic DNA (gDNA) necessary for long-read sequencing was extracted using the Blood & Cell Culture DNA Mini Kit-G20 (Qiagen) and 700 ng and 1 µg of DNA was used for PacBio and Nanopore library preparation, respectively (Fig. 1B). HMW PacBio libraries were generated following the Pacific Biosciences protocol “Preparing HiFi SMRTbell® Libraries using SMRTbell Express Template Prep Kit 2.0”. The libraries were run on an 8 M SMRTCell using sequencing reagents version 2.0. Sequencing was performed on a Sequel II sequencer (Pacific Biosciences) with control software version 9.0.0.9223 and a movie collection time of 15 hours per SMRTCell. ONT libraries were generated using the Ligation Sequencing SQK-LSK109 kit protocol per manufacturer’s instructions (Nanopore-MinION) and sequencing was performed on a FLO-MIN106 flow cell using the operation software MinkNOW (version 21.06.9) for 60 hours. In total 947,266 and 2,631,769 raw reads were generated from PacBio and ONT sequencing, respectively (Table 1). PacBio reads were used for genome assembly and ONT reads larger than 10 kb were used for scaffolding. Total RNA was extracted using RNeasy Micro Kit (Qiagen) and submitted to CD Genomics (New York, USA) for library preparation and sequencing. RNA libraries were prepared using SMART-Seq v4 Ultra Low Input RNA Kit (Takara) and sequencing was performed using Hi-Seq X Ten (Illumina) by CD Genomics. A total of 55,879,424 cleaned reads were used for genome annotation (Table 1).

**Table 1:** Sequencing data statistics.

Platform	Molecule	Step	Average length (bp)	Number of raw reads	Number of cleaned reads	BioProject accession
PacBio - Sequel II	DNA	Assembly	8,937.87	947,266	NA	PRJNA841657
Nanopore- MinION	DNA	NA	6,461.50	2,631,769	NA	PRJNA841657
Nanopore- MinION reads >10 kb	DNA	Scaffold	16,875.40	317,216	NA	NA
Illumina - Hiseq	RNA	Annotation	150	56,448,514	55,879,424	PRJNA841657

NA - Not Applicable.

### Genome assembly

PacBio HiFi reads were assembled using canu (v2.1)^9^. MinION reads >10 kb were used to correct and scaffold the contigs using LongStitch (v 1.0.1)^10^ with the parameters k = 32, w = 250, with the gap fill option selected. Next, the assembly was polished using the PacBio reads with racon (v1.4.13)^11^. Genomic summary statistics were determined using Galaxy^12^. Forty contigs were removed from the assembly due to being <1 kb in length or having either zero or only one MinION read mapping to the PacBio assembly. Only contigs that had similar coverage from both long-read sequencing platforms were included. The genome had a GC content of 29.47% (Table 2). In summary, the 193.49 Mbp genome was assembled in 756 contigs with N50 of ~ 3.5 Mb, the shortest contig size was 7,256 bp and the longest was ~11 Mbp (Table 2).

**Table 2:** *T.musculus* genome assembly statistics.

Genome assembly length (Mbp)	193.49
Number of contigs	756
N50	3,579,227
L50	15
N90	305,259
Shortest contig	7,257
Longest contig	11,419,247
G + C content (%)	29.47
Portion covered by repetitive regions	42.67%

### Gene and functional predictions

Gene annotation was done using funannotate (v1.8.11)^13^ which removed 298 duplicate contigs from the assembly. Repeat masking was done using redmask (v0.0.2)^13^ which masked 42.67% of the genome, indicating that it is a highly repetitive genome, which likely explains its larger size compared to other trichomonads (Table 3). NCBI’s Foreign Contamination Screen^14^ identified 18 contigs as contaminants that were removed from the assembly and an additional 4 contigs as chimeric that were separated into multiple contigs with the intervening sequence removed. RNA-Seq data used by funannotate was assembled with Trinity (v2.12.0)^15^. Funannotate ran the following programs: PASA (v2.5.2)^16^, GeneMark (v4)^17^, Augustus (v3.3.3)^18^, eggnog-mapper (v2.1.2)^19^, signalp (v6)^20^, and interproscan (v5.52–86.0)^21^. Ribosomal RNA genes were identified by RNAmmer (v1.2)^22^. Relatively few genes possessed introns (5.66%) and the gene function annotation predicted that 36.6% of the genome was covered by 46,131 genes. BUSCO statistical analysis showed that the genome assembly captured 100% of the expected trichomonad genes, indicating that the genome assembly was complete, but only 53% of available BUSCO genes within the eukaryote_odb10 database (Table 4). Accordingly, 44,152 genes were identified as protein coding genes and 39,744 as hypothetical genes. Of these, 24,105 of the hypothetical genes have an InterPro number, 13,990 have a GO (Gene Ontology) term, 4,671 have an EC (Enzyme Commission) number and 24,215 have Interpro, GO and EC numbers (Table 3).

**Table 3:** *T. musculus* genome annotation statistics.

Portion of genome covered by genes	36.60%
Number of genes	46,131
Number of CDS	44,152
Number of genes with introns	2,500
Number of tRNA transcripts	1,738
Number of 5S rRNAs	218
Number of 18S rRNAs	12
Number of 28S rRNAs	11
Number of protein coding genes	44,152
Number of non protein coding genes	1,979
Number of hypothetical genes	39744
Number of hypothetical genes with InterPro number	24105
Number of hypothetical genes with Gene Ontology (GO)	13990
Number of hypothetical genes with Enzyme Commission (EC)	4671
Number of hypothetical genes with InterPro, GO, or EC	24215

**Table 4:** BUSCO statistics.

Complete BUSCOs (C)	53%
Complete and single-copy BUSCOs (S)	41.60%
Complete and duplicated BUSCOs (D)	11.40%
Fragmented BUSCOs (F)	7.10%
Missing BUSCOs (M)	39.90%
Total BUSCO groups searched	255

### Ploidy analysis

**Fig. 2:**
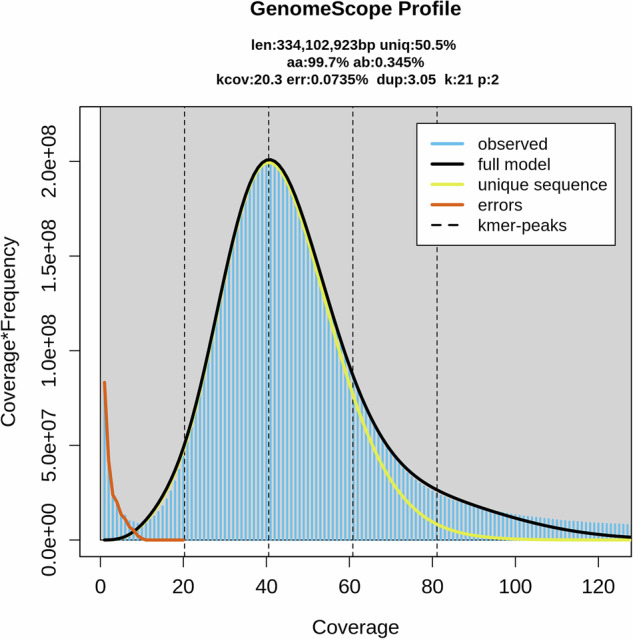
GenomeScope plot visualization of *T.mu* genome presented a single k-mer peak, indicated haploid genome. K-mer analysis (k = 21) estimated genome size is 334 Mb with coverage of 20.3X, 50.5% unique sequences, homozygous rate of 99.7% and heterozygosity rate of 0.345%.

Ploidy analysis was performed using a combination of GenomeScope 2.0 and Smudgeplot^23^ using *T. musculus* Illumina Hi-Seq reads (150 bp, two biological replicates). GenomeScope was run to fit a mixed model of negative binomial distributions to the k-mer spectrum of sequencing data as measured by FASTK (Fig. 2). The k-mer spectra was also visualized using Smudgeplot to estimate organism ploidy. The ploidy analysis by GenomeScope was consistent with *T. musculus* being haploid, with a homozygosity rate of 99.7% and heterozygosity rate of 0.345% (Fig. 1). However, the ploidy analysis using Smudgeplot (Sup Fig. 1), a software suite trained to recognize polyploid states, rather supported an organism that was diploid with highly homozygous sister chromosomes.

### Comparative functional genomic analysis

**Fig. 3:**
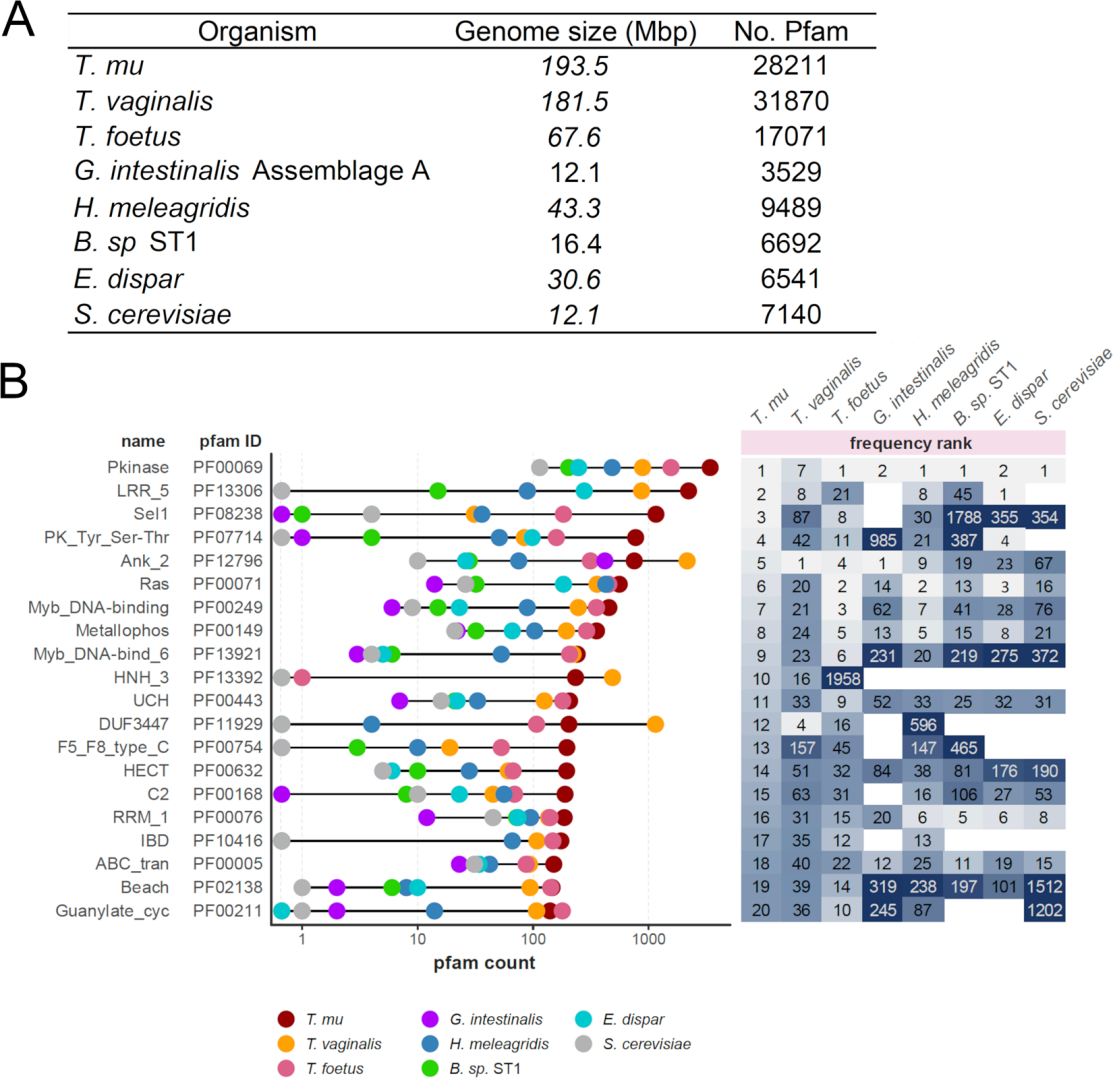
Functional protein domain comparison between *T. musculus* and other protists colonizing the mammalian gut mucosa. (**A**) Total number of Pfam domains identified for each annotated protist genome analyzed. *Saccharomyces cerevisiae* genome was used as a model organism. (**B**) Top 20 most abundant Pfam domains detected for all annotated *T. musculus* protein sequences, ranked by their frequency of occurrence compared to other protists and yeast (*left panel*). Each unique Pfam domain was ranked by the frequency of its occurrence. The number indicated in each box represents the rank order within each species from most abundant to least abundant (*right panel*). Shading was dependent on rank order, with darker shades indicating a lower rank.

A gene functional comparison of *T. musculus* was performed by comparing predicted Pfam domains against other representative genomes, including protozoa that colonize the mammalian gut mucosa and are described as pathogenic or opportunistic, as well as *Saccharomyces cerevisiae*. Predicted *T. musculus* proteins were submitted to InterProScan v.5.30–69.0^21^ for functional annotation (minimum E-value 1e-05). Pfam domains were retrieved from available annotated genomes for *Trichomonas vaginalis* G3 2022, *Tritrichomonas foetus* strain K, *Giardia* Assemblage A isolate WB 2019 (GiardiaDB release 62), *Histomonas meleagridis* 2922-C6/04-10x (TrichDB release 62) and *Entamoeba dispar* SAW760 (AmoebaDB release 62). For *Blastocystis* sp. ST1 (UP000078348) and *Saccharomyces cerevisiae* (UP000002311), Pfam annotations were retrieved from proteomes in UniProt release 2023_01 (Fig. 3A). Overall frequencies of domain-containing proteins were tabulated and ranked for each organism.

We examined the frequency of the 20 most abundant Pfam domains identified in the *T. musculus* proteome against other infectious protists and *S. cerevisiae* (Fig. 3B; Suppl. Table 1). The most prevalent domain in *T. musculus*, the protein kinase domain (PF00069), with 3411 copies in the genome, was consistent with other organisms. As expected, the transcription-initiator DNA-binding domain IBD (PF10416), first reported to bind a DNA element unique to *T. vaginalis*, was present only in the trichomonads^24^. Of particular interest was the BspA-type Leucine rich repeat region (LRR_5 PF13306) found in 2,217 *T. musculus* proteins. BspA-like proteins are known to mediate interactions with the host extracellular matrix and were previously reported in large numbers in *T. vaginalis* and *Entamoeba* spp^25,26^. We also identified an expansion in the multi-antimicrobial extrusion protein domain (MatE; PF01554) in trichomonads (87 genes in *T. musculus*, 36^th^ most abundant domain; 48 and 44 genes in *T. vaginalis* and *T. foetus*; 14 genes in *H. meleagridis*), whereas the domain was detected in only three or fewer genes among the remaining protists, and yeast.

## Data Records

The *T. musculus* genome assembly (isolate EAF2021) and raw reads have been deposited in the NCBI database under the BioProject accession number PRJNA841657, SRA accession numbers SRR25145092, SRR25067786, SRR25067787^27^, GenBank accession JAPFFF000000000.1^28^ and GeneBank assembly GCA_039105265.1^29^.

## Technical Validation

Benchmarking Universal Single-Copy Orthologs (BUSCO) is a comprehensive tool to evaluate the quality of the genome assemblies and transcriptomes for eukaryotes and prokaryotes^30,31^. To estimate the completeness of the *T. musculus* assembly, we conducted BUSCO (version 5.4.2) analyses using mode euk_genome_met mode and metaeuk as gene predictor (eukaryote_odb10). BUSCO reported 53% total complete BUSCO genes representing 132 out of 225 BUSCOs and 104 complete and single-copy BUSCOs (41.6%) (Table 4). BUSCOs scores lower than 50% have been observed in published protists genomes^32–34^ and they are acceptable as satisfactory and indicate a good completeness. The low number detected is likely due to the lack of complete or partial BUSCO datasets for protists, especially for *T. musculus* related species.

## Supplementary information

Supplementary Figure 1

Supplementary Table 1

Supplementary Figure 1

## Data Availability

No custom codes were used in this study. All bioinformatics tools were used following the public access instructions detailed in the methods section.
