# Supplementary figures and images for: Whole Genome Sequence of the gut commensal protist Tritrichomonas musculus isolated from laboratory mice
Source: Sci Data. 2025 Apr 8;12:590. doi: 10.1038/s41597-025-04921-0 (PMC11978803; doi:10.1038/s41597-025-04921-0)

*T.musculus*  
Proposed diploid

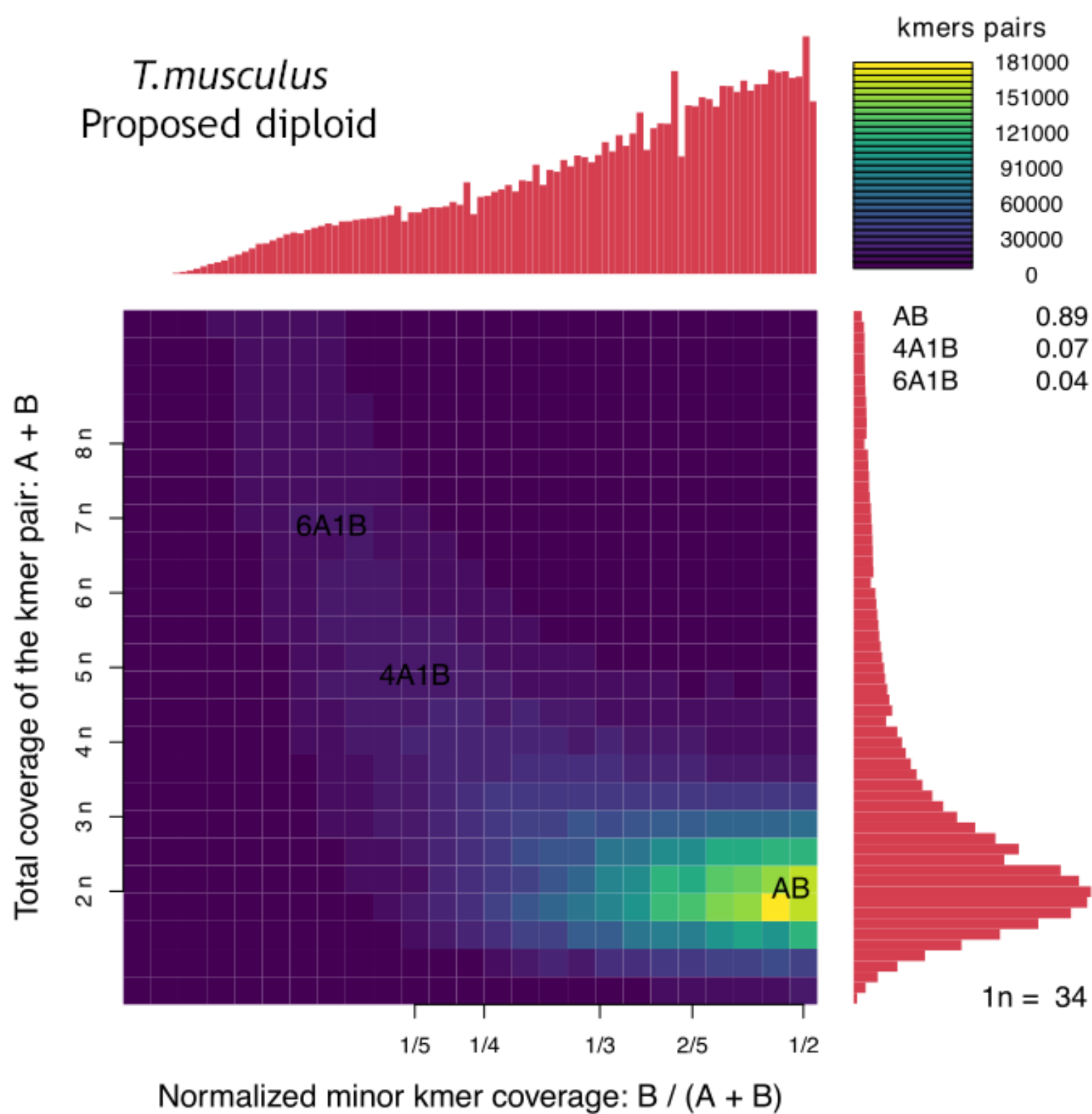

Supplement: Supplementary file 1 — Supplementary Figure 1 [file 41597_2025_4921_MOESM1_ESM.pdf]
