# Supplementary material for: Whole Genome Sequence of the gut commensal protist Tritrichomonas musculus isolated from laboratory mice
Source: Sci Data. 2025 Apr 8;12:590. doi: 10.1038/s41597-025-04921-0 (PMC11978803; doi:10.1038/s41597-025-04921-0)
Supplement: Supplementary file 3 — Supplementary Figure 1 [file 41597_2025_4921_MOESM3_ESM.docx]

**Supplementary Figure 1. Visualization of k-mer spectra using Smudgeplot suggests that *T.musculus* is either haploid or largely homozygous diploid.** Smudgeplot indicates an enrichment of k-mer abundance around the AB coordinates of the CovB/(CovA+CovB) plot and no other areas of k-mer enrichment, suggesting that *T.musculus* is likely haploid or largely homozygous diploid.

**Supplementary Table 1. *T. musculus* Pfam domain frequency ranking with domain descriptions**
